# Carotid body physiology meets cytochrome c oxidase crystallography Commentary to Ortega-Sáenz P, López-Barneo J. Physiology of the Carotid Body: From Molecules to Disease. *Annu Rev Physiol* 82: 127–149, 2020. Torres-Torrelo H, Ortega-Sáenz P, Gao L, López-Barneo J. Lactate sensing mechanisms in arterial chemoreceptor cells. *Nat Commun* 12: 4166, 2021

**DOI:** 10.1007/s00424-021-02662-8

**Published:** 2022-01-04

**Authors:** Helmut Acker, Joachim Fandrey

**Affiliations:** Institut Für Physiologie, Universität Duisburg-Essen, Hufelandstr. 55, D-45147 Essen, Germany

The carotid body (CB) oxygen-sensing mechanism remains elusive. Mitochondrial complex I and complex IV of CB type I cells are the most likely oxygen sensor candidates for initiating the hypoxia-induced release of neurotransmitters (NT) to excite synaptically connected sinus nerve fibers [7]. CB cytochrome c oxidase (CBCcO) is specialized by the subunit COX 4i2 [2, 7] which is characterized by twofold lower oxygen affinity than COX 4i1 [8]. In general, CcO contains four redox centers: heme a and heme a3 linked by helix-X and two copper centers (CuA and CuB). Oxygen binds to the heme a_3_-Cu_B_ binuclear center as described in crystallographic studies on bovine CcO (bCcO) microcrystals [3]. When helix-X relaxes, communication between the two heme groups facilitates electron transfer from mitochondrial cytochrome c (complex III) over CuA to heme a and the binuclear center.

**Fig. 1:**
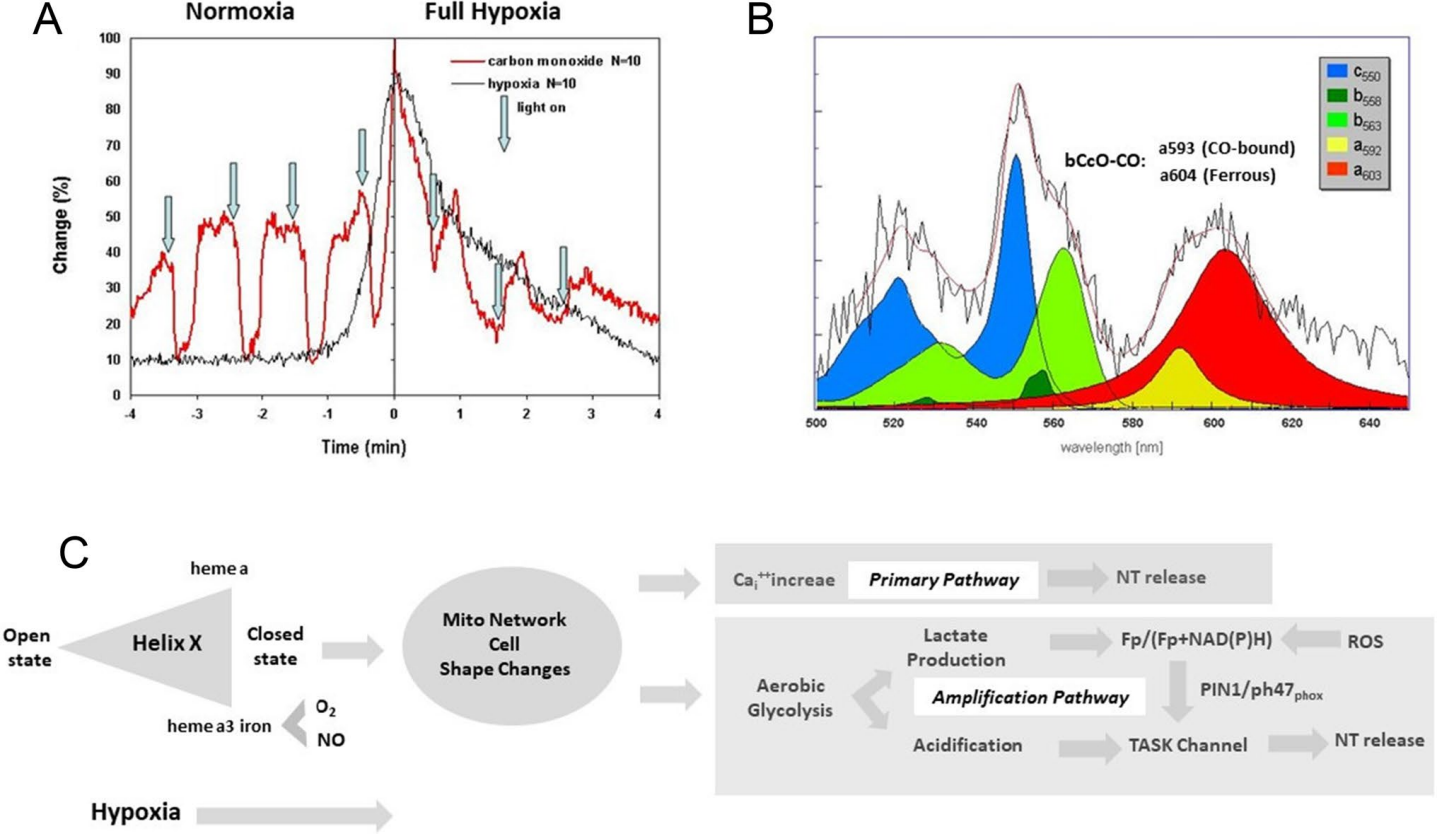
CcO crystallographic changes of about 3 Ångström are displayed by CB chemoreceptor discharge. **a** Chemoreceptor discharge under normoxic and hypoxic CO application (red registration line) The effect of CO is shown as a percentage of peak chemoreceptor activity induced by 4-min hypoxia. Control chemoreceptor discharge without CO is shown as the black line. Data are mean values from 10 carotid bodies. CO application during normoxia (left side at time − 4) leads to excitation, which is eliminated during light-induced photodissociation (vertical arrows). CO application during full hypoxia (right side of time zero) leads to discharge inhibition, which is reversed during photodissociation [4]. **b** Identification of carotid body heme proteins by light absorption photometry. N_2_ versus aerobic steady-state spectrum (black solid noisy line; mean values of 6 carotid bodies) was fitted by different mitochondrial and non-mitochondrial cytochrome spectra as indicated by different colors. The deconvolution fit curve (red solid line) obtained by varying the amplitude of the optical density of five cytochromes closely fits the experimental curve [1]. CBCcO double-absorption peaks are assumed to be comparable to bCcO CO bound and ferrous absorption peaks. **c** Changes in Helix-X from open to closed state under hypoxia are fine-tuned by interaction of O_2_ and NO binding on heme a3 iron. Subsequent mitochondria-induced cell shape changes lead to intracellular calcium increase and NT release (primary pathway). Reduced electron transfer between heme a and heme a3 stimulates aerobic glycolysis with subsequent lactate production. Subsequent tissue acidification inactivates TASK channels. In addition, ROS affecting the redox status involving PIN1/p47_phox_ interact with TASK channels (amplification pathway) [2]

As surrogate for oxygen, CO and NO replace oxygen at the binuclear center in a light-sensitive way and inhibit electron transfer [3]. Upon light-induced CO photodissociation under reducing conditions, helix-X switches from the open to the closed state when ferrous heme a3 iron is in an exogenous ligand-free state like in hypoxia [3]. Based on these studies, we present a deeper understanding of the nature of the CB oxygen sensor by reanalyzing CO experiments on isolated rat CBs super-fused in vitro as shown in Fig. 1a [4]. Changing gas composition of the super-fusion medium from 20% O_2_, 3% CO_2_, 77% N_2_ (normoxia) to 97% N_2_, 3% CO_2_ (full hypoxia) is followed by a characteristic hypoxia-induced increased chemoreceptor sinus nerve activity (registration in black) serving as control. Replacing 77% N_2_ by CO induces an increased sinus nerve activity (registration in red) which is decreased to normoxic control levels by CO photo-dissociation and oxygen ligand binding to the binuclear a3-CuB center reestablishing electron transfer. Additional CO to fully replace O_2_ (resulting in 97% CO and 3% CO_2_) increases chemoreceptor sinus nerve activity (full hypoxia, registration in red). CO photo-dissociation is followed by a further increase of chemoreceptor discharge reaching hypoxic control levels. We propose that ferrous heme a3 iron now is in an exogenous ligand-free state and helix-X compressed into a closed conformation with inhibited electron transfer due to increased distance between heme a/a3.

We assume this large helix-X movement of about 3Ǻ [3] acts as a primary sensor signal to trigger chemoreceptor discharge under hypoxia by inducing a motion of the intracellular mitochondrial network [10] with subsequent cell shape changes (see Fig. 1c). This motion might trigger mechano-sensitive cation membrane channels leading to a primary rise of intracellular calcium initiating NT release.

To further characterize CBCcO, the mean hypoxic light absorption spectrum (black registration) of CBCcO was recorded (Fig. 1b; [1, 4]). The spectrum was fitted by deconvolution (red line) using characteristic light absorption spectra of mitochondrial cytochromes (c550, b563) and NADPH cytochrome (b558). CBCcO peaks at 592 nm and 603 nm matching light absorption spectra of bCcO-CO microcrystals peaking at a593nm (CO-bound) and a604nm (ferrous) [3]. We assume that the CBCcO double peak results from binding of NO produced under hypoxia [6]. NO inhibits hypoxic CB chemoreception, while it increases the chemosensory discharges in normoxia [6]. Upon binding to CBCcO, NO seems to induce the same helix-X changes like CO. NO ligand binding to CBCcO is reported to decrease oxygen consumption and favoring aerobic glycolysis [2]. Subsequent tissue acidification and lactate production as shown in Fig. 1c could lead to amplification of the primary calcium signal by closing TASK channels involving the PIN1/p47_phox_ tandem [2] and influencing the Fp/(Fp + NAD(P)H) redox ratio [2, 5, 9].
